# When similar is not the same: sex-specific outcomes and risk factors in thoracoabdominal aortic repair

**DOI:** 10.3389/fcvm.2025.1734089

**Published:** 2026-01-16

**Authors:** Florian Helms, Heike Krüger, Ezin Deniz, Alina Botezatu, Andreas Martens, Aron-Frederik Popov, Stefan Rümke, Bastian Schmack, Jan Dieter Schmitto, Alexander Weymann, Arjang Ruhparwar, Morsi Arar

**Affiliations:** 1 Division for Cardiothoracic, Transplantation and Vascular Surgery, Hannover Medical School, Hannover, Germany; 2 Clinic for Cardiac Surgery, University Clinic Oldenburg, Oldenburg, Germany; 3 Clinic for Cardiac Surgery, Asklepios Clinic Harburg, Hamburg, Germany

**Keywords:** aortic aneurysm, aortic surgery, individualized therapy, sex-specific analysis, thoracoabdominal aorta

## Abstract

**Objective:**

Growing evidence suggests major outcome and risk factor disparities between men and women undergoing cardiovascular surgery. Thus, sex-specific approaches are increasingly being adopted in cardiovascular medicine. However, data on sex-specific outcomes and risk stratification in complex thoracoabdominal aortic repair remain limited.

**Methods:**

We present a retrospective single-center analysis of 311 consecutive patients, including 99 women (31.8%), who underwent open surgical thoracoabdominal aortic repair between 2000 and 2024. Propensity score matching was performed prior to a comparative analysis of intraoperative parameters, postoperative outcome, and complications, as well as short- and long-term mortality between female and male patients.

**Results:**

In the initial study population, men had a significantly higher BMI (26.3 vs. 23.1 kg/m^2^, *p* < 0.001) and greater prevalence of coronary artery disease (37.7% vs. 21.2%, *p* = 0.004) and hyperlipidemia (27.8% vs. 12.1%, *p* = 0.002) compared to women. Postoperatively, wound infections were more frequent in women in the unmatched cohort (12.1% vs. 4.3%, *p* = 0.01), but no sex-related differences in mortality, ICU length of stay, or long-term survival were observed after propensity score matching. Multivariate regression revealed highly distinct predictors of early mortality in each sex: prior cardiac surgery and urgency in men versus hypertension, chronic kidney disease, coronary artery disease, and older age at the time of operation in women.

**Conclusion:**

Overall outcomes and survival following thoracoabdominal aortic repair were comparable between men and women. However, underlying risk factors for early mortality differed fundamentally between sexes. These findings underscore the importance of a sex-specific preoperative risk assessment in the surgical decision-making process prior to open thoracoabdominal aortic repair.

## Introduction

In recent years, the recognition of sex-specific disparities in cardiovascular medicine has become increasingly important. Increasing evidence demonstrates that men and women differ significantly in disease presentation, progression, treatment response, and postoperative outcomes across various cardiovascular conditions (1, 2). In cardiac surgery, female patients often present with more comorbidities and face higher perioperative risks, including increased mortality and prolonged hospital stays (3, 4). Despite these well-documented disparities, clinical guidelines and risk scores largely remain sex-neutral, failing to incorporate female-specific physiological and anatomical considerations. In aortic surgery, sex-related biological and clinical differences may significantly affect outcomes. However, recommendations and thresholds for aortic surgery indication rarely account for sex-specific differences (5).

Emerging data highlight important sex-specific outcome differences in proximal aortic surgery. For instance, a retrospective study by van Kampen et al. of 1,773 patients undergoing ascending aortic replacement procedures found that women had significantly higher in-hospital mortality (3.6% vs. 0.9%, *p* < 0.001), with longer postoperative ventilation and ICU length of stays (6). Similarly, a large-scale matched analysis of ascending and arch procedures reported vast discrepancies in the preoperative risk factors for postoperative adverse events for women compared to men, despite similar early mortality and complication rates (7). The findings suggest that the current universal model used for surgical decision-making in thoracic and thoracoabdominal aortic surgery neglects female-specific risk profiles. Without sex-specific cutoffs, timing criteria, or surgical thresholds, women may face suboptimal perioperative care and elevated complication rates.

Knowledge regarding sex-specific risk profiles in thoracoabdominal aortic aneurysm repair is even more limited, with very few studies published by high-volume centers: In a larger retrospective study, Spiliotopoulos et al. found no significant differences in short-term postoperative outcomes and early mortality between men and women, although perioperative risk factors for early mortality differed between the sexes (8). The impact of sex-specific preoperative patient characteristics and burden of comorbidities on postoperative outcomes after open thoracoabdominal aortic repair still remains unknown. However, this information about the influence of patient characteristics is critical for practicing surgeons and physicians in tailoring therapeutic strategies to individual patients. This represents a significant knowledge gap, particularly given the disproportionately high comorbidity burden observed in female patients.

Therefore, we present a single-center analysis comparing sex-specific rates of postoperative complications as well as short- and long-term survival. Furthermore, we provide a sex-specific analysis of preoperative risk factors for early mortality in women versus men undergoing open surgical thoracoabdominal aortic repair.

## Methods

### Patients and data collection

A total of 311 consecutive patients who underwent open surgical thoracoabdominal repair at our institution between 2000 and 2024 were included in the study. Among these, 68.2% (*n* = 212) were male and 31.8% (*n* = 99) were female. Preoperative patient characteristics, comorbidity burden, and extent of disease, as well as intraoperative parameters and operation times, postoperative complications, length of intensive care unit and hospital stay, and short-term mortality, were collected prospectively in our institutional database and analyzed retrospectively. If missing values were discovered during the retrospective evaluation, they were added using the clinic's internal documentation and archiving system to ensure completeness of the data. Long-term follow- up was performed via our aortic outpatient clinic with regularly scheduled follow-up visits. Data collection and analysis were conducted in accordance with ethics board approval by the Ethics Commission of Hannover Medical School (Approval No. 11461_BO_K).

### Study design

**Figure 1:**
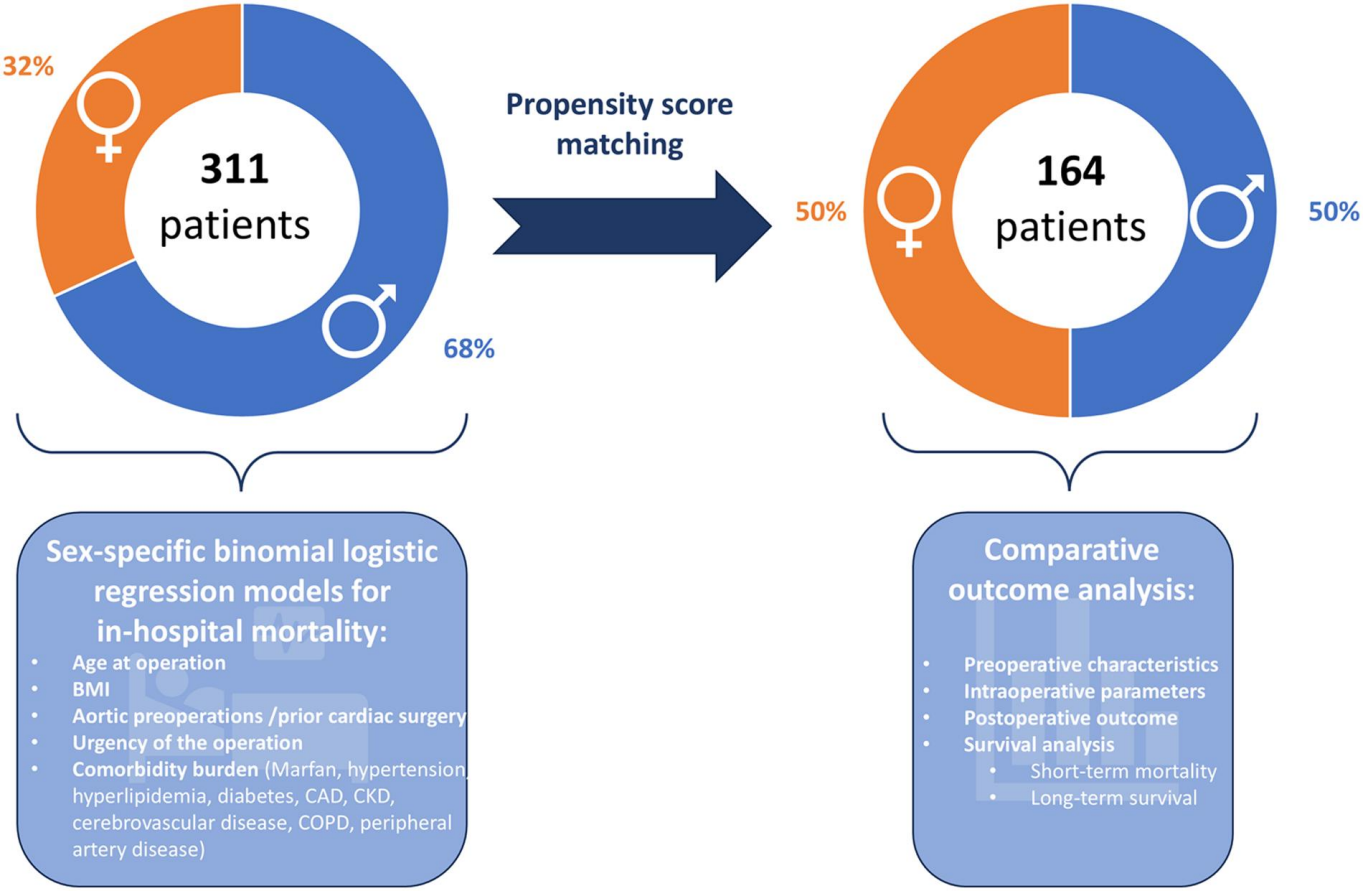
Study design. The study was conducted in two steps: First, propensity score matched analysis of pre-, intra-, and postoperative parameters of women versus men undergoing open surgical thoracoabdominal aortic repair was performed. Second, sex-specific binominal regression models for in-hospital mortality were generated and analyzed for predicting variables. Icons from Microsoft 365.

This retrospective single-center analysis with prospective data collection and follow up was performed using a two-step approach (Figure 1). In the first step, one-to-one propensity score matching of female and male patients undergoing thoracoabdominal aortic repair was conducted. For this, groups were adjusted for discrepancies in preoperative characteristics such as age at operation and body mass index, as well as comorbidity burden including cardiovascular, respiratory, renal, and neurological comorbidities. The full list of preoperative parameters used for propensity score matching is provided in Supplementary Table S1. Patients’ sex was defined as the group indicator for propensity score matching. A logistic regression was performed on the group indicator, and the resulting propensity variable was used to select controls for the demander cases. The caliper was set to 0.05. A 1:1 propensity score matching without replacement was performed. Unmatched cases were excluded from outcome analyses in the matched cohort. Following this, both the initial study population and the matched groups were compared for pre-, intra-, and postoperative characteristics and outcome parameters. Comparative analyses between groups and survival analyses were performed for both the unmatched and the matched cohorts in parallel to account for matching and potential differences resulting from unequal baseline values.

In the second step, a multivariate binomial logistic regression model for in-hospital mortality was created separately for female and male thoracoabdominal aortic repair patients. Preoperative patient characteristics, comorbidities, and urgency of the operation were used as predicting variables. The full list of tested predicting variables is provided in Supplementary Table S2. Linearity was tested using the Box–Tidwell method with Bonferroni correction for all continuous variables (9). Goodness of fit of the logistic regression model was analyzed using the Hosmer–Lemeshow Test. To investigate possible interactions among the predicting variables, a combined model of the original study population was generated, incorporating the key predictors identified in the separate models. A correlation matrix was then constructed to identify possible interactions between predictors.

### Surgical technique

The surgical technique applied for open surgical thoracoabdominal aortic replacement did not differ between female and male patients. In both groups, thoracoabdominal repair was performed as described previously (10). Briefly, patients were placed in hemi-right-sided position, and a left-sided thoracotomy starting in the 5th to 7th intercostal space was performed and extended depending on the location and extension of the aortic pathology. The incision was then extended toward a paramedian laparotomy to access the abdominal aorta. After establishing cardiopulmonary bypass and cooling, the aorta was clamped proximally and distally, thus isolating the aneurysmatic segment, and graft implantation was performed according to the extent of the aortic disease. Intercostal, lumbar, and visceral arteries were reimplanted when required and suitable. After rewarming, deairing, weaning from cardiopulmonary bypass, and achieving hemostasis, patients were transferred to the surgical intensive care unit for further stabilization. To identify potential bias due to practice changes over the inclusion period, time frame-stratified sex distribution between male and female patients operated over time was analyzed.

### Study definitions

The extent of thoracoabdominal aortic repair was categorized according to the Crawford classification of aortic diseases, considering the proximal and distal prosthesio-aortic anastomosis for sorting in the corresponding group (11). Cerebrovascular disease was defined as any radiographically diagnosed flow-limiting intra- or extracranial cerebrovascular stenosis or occlusion. Likewise, peripheral vascular disease was defined as any clinically radiographically diagnosed flow-limiting peripheral vascular stenosis or occlusion. Coronary artery disease was defined as any treated or untreated flow-limiting coronary artery obstruction diagnosed by coronary angiography. Chronic kidney disease was defined as a decreased glomerular filtration rate of less than 60 mL/min (1.73 m^2^) for at least 3 months (12). Acute kidney failure was defined as a threefold increase in serum creatinine or urine output of less than 0.5 mL per kg body weight per hour for 24 h (13). Sepsis was defined according to the Third International Consensus Definitions for Sepsis and Septic Shock (Sepsis-3) (14). Low cardiac output syndrome was defined as a cardiac index <2.2 L/min and body surface area in m^2^ with systolic blood pressure of <90 mmHg or recurrent catecholamine dependency. Respiratory failure was defined as the necessity of re-intubation or non-invasive ventilation after previous spontaneous breathing. Symptoms and complications resolving prior to discharge were defined as temporary, while those persisting at discharge were defined as permanent. Operations were categorized as urgent if the time between first admission and operation was under one week and as emergent if the time between first admission and operation was under 24 h.

### Statistics

Statistical analyses were performed using IBM SPSS Statistics 29 (IBM Corp., Armonk, NY, USA, 1989, 2021). Normal distribution of continuous variables was tested using the Shapiro–Wilk test. Non-normally distributed data are presented as median and interquartile range. The Mann–Whitney test was used for comparison of non-normally distributed data. A *p*-value of <0.05 was considered significant. Long-term survival analysis was performed for female and male patients in both the matched and unmatched cohort using Kaplan–Meier survival estimates and log-rank testing.

## Results

### Patient profile and propensity score matching

A total of 311 consecutive patients, including 99 women (31.8%), were included in the initial study population. Prior to propensity score matching, male patients had significantly higher body mass indices (BMI) with an index of 26.3 (IQR 24.3–28.9) in men compared to 23.1 (IQR 20.2–27.4) in women (Table 1). Furthermore, the male patients had higher rates of metabolic disorders, with significant differences in the preoperative prevalence of hyperlipidemia [12.12% (*n* = 12) vs. 27.83% (*n* = 59), *p* = 0.002] and coronary artery disease [21.21% (*n* = 21) vs. 37.74% (*n* = 80), *p* = 0.004] between male and female patients at the time of open thoracoabdominal aortic repair. In the time-stratified sex distribution analysis, the proportion of female and male patients operated on over time remained stable across all periods examined, ranging between 25% and 36% (Supplementary Figure S1).

**Table 1:** Preoperative characteristics.

Characteristics	Unmatched cohort					Matched cohort				
	Overall	Female	Male	*p*-Value	SMD	Overall	Female	Male	*p*-Value	SMD
Age at operation	62 (52–68)	64 (53–70)	60 (52–67)	0.051	1.43	64 (53–70)	63 (52–70)	64 (55–70)	0.968	−0.27
BMI	25.5 (22.8–28.1)	23.1 (20.2–27.4)	26.3 (24.3–28.9)	**<0** **.** **001**	1.14	24.4 (21.9–27.5)	23.9 (20.6–27.7)	24.7 (22.6–27.1)	0.201	0.22
Marfan	12.86% (*n* = 40)	16.16% (*n* = 16)	11.32% (*n* = 24)	0.275	1.12	13.41% (*n* = 22)	15.85% (*n* = 13)	10.98% (*n* = 9)	0.493	0.91
Hypertension	65.27% (*n* = 203)	59.6% (*n* = 59)	67.92% (*n* = 144)	0.161	−1.41	64.63% (*n* = 106)	63.41% (*n* = 52)	65.85% (*n* = 54)	0.870	−0.32
Hyperlipidemia	22.83% (*n* = 71)	12.12% (*n* = 12)	27.83% (*n* = 59)	**0** **.** **002**	−3.49	18.29% (*n* = 30)	14.63% (*n* = 12)	21.95% (*n* = 18)	0.313	−1.27
Diabetes	5.14% (*n* = 16)	2.02% (*n* = 2)	6.6% (*n* = 14)	0.088	−2.07	1.83% (*n* = 3)	2.44% (*n* = 2)	1.22% (*n* = 1)	0.560	−0.58
Coronary artery disease	32.48% (*n* = 101)	21.21% (*n* = 21)	37.74% (*n* = 80)	**0** **.** **004**	−3.13	29.88% (*n* = 49)	24.39% (*n* = 20)	35.37% (*n* = 29)	0.172	−1.55
Cerebrovascular disease	8.36% (*n* = 26)	9.09% (*n* = 9)	8.02% (*n* = 17)	0.750	0.31	10.98% (*n* = 18)	7.32% (*n* = 6)	14.63% (*n* = 12)	0.211	−1.51
Chronic renal disease	19.94% (*n* = 62)	16.16% (*n* = 16)	21.7% (*n* = 46)	0.255	−1.19	17.07% (*n* = 28)	14.63% (*n* = 12)	19.51% (*n* = 16)	0.534	−0.83
COPD	15.11% (*n* = 47)	12.12% (*n* = 12)	16.51% (*n* = 35)	0.314	−1.10	16.46% (*n* = 27)	14.63% (*n* = 12)	18.29% (*n* = 15)	0.674	−0.63
Tobacco smoking	22.51% (*n* = 70)	20.2% (*n* = 20)	23.58% (*n* = 50)	0.506	−0.67	17.68% (*n* = 29)	17.07% (*n* = 14)	18.29% (*n* = 15)	0.838	−0.20
PVD	15.11% (*n* = 47)	8.08% (*n* = 8)	18.4% (*n* = 39)	0.018	−2.70	12.2% (*n* = 20)	8.54% (*n* = 7)	15.85% (*n* = 13)	0.232	−1.45
Aortic preoperation	50.5% (*n* = 157)	43.4% (*n* = 43)	53.8% (*n* = 114)	0.089	−1.72	46.3% (*n* = 76)	46.3% (*n* = 38)	46.3% (*n* = 38)	1.000	0

SMD, standardized mean differences; BMI, body mass index; COPD, chronic obstructive pulmonary disease; PVD, peripheral vascular disease.

Statistically significant values are highlighted in bold.

Propensity score matching identified 82 one-to-one pairs of men and women from the initial study cohort, yielding a total of 164 patients, representing 52.7% of the initial study population and including 82.8% of the female patients in the study. The indication for surgery for the vast majority of patients, in both the male and female groups, was aneurysm of the aorta. Chronic dissection was present in 37.8% (*n* = 31) of the male patients and 30.5% (*n* = 25) of the female patients in the matched patient cohort (*p* = 0.410). In addition, one female patient (1.2%) was operated upon for late-diagnosed coarctation of the aorta. Through propensity score matching for the preoperative characteristics listed in Supplementary Table S1, adjustment for the differences in BMI and burden of disease between men and women in the unmatched cohort was achieved so that no statistically significant differences were found between the two groups in the propensity score matched cohort (Table 1). In the matched cohort, the standardized mean differences (SMD) between female and male patients were smaller than in the unmatched study population for all preoperative variables, except cerebrovascular disease.

### Intraoperative parameters

Operative urgency did not differ between male and female patients in either the unmatched or the matched cohort (Table 2). In the unmatched cohort, a significantly higher incidence of Crawford Type 1 operations was performed in the female subgroup [18.37% (*n* = 18) vs. 9.48% (*n* = 20), *p* = 0.027], while no significant differences in the Crawford extents of repair were found after propensity score matching. Likewise, operation time, bypass time, and cross-clamp time did not differ significantly between the two groups.

**Table 2:** Intraoperative characteristics.

Characteristics	Unmatched cohort				Matched cohort			
	Overall	Female	Male	*p*-Value	Overall	Female	Male	*p*-Value
Urgent	6.43% (*n* = 20)	4.04% (*n* = 4)	7.55% (*n* = 16)	0.240	3.05% (*n* = 5)	3.66% (*n* = 3)	2.44% (*n* = 2)	0.650
Emergent	8.04% (*n* = 25)	9.09% (*n* = 9)	7.55% (*n* = 16)	0.641	7.32% (*n* = 12)	6.1% (*n* = 5)	8.54% (*n* = 7)	0.766
Crawford extent of repair I	12.3% (*n* = 38)	18.37% (*n* = 18)	9.48% (*n* = 20)	**0** **.** **027**	15.85% (*n* = 26)	17.07% (*n* = 14)	14.63% (*n* = 12)	0.831
Crawford extent of repair II	22.33% (*n* = 69)	23.47% (*n* = 23)	21.8% (*n* = 46)	0.743	24.39% (*n* = 40)	25.61% (*n* = 21)	23.17% (*n* = 19)	0.856
Crawford extent of repair III	38.19% (*n* = 118)	33.67% (*n* = 33)	40.28% (*n* = 85)	0.266	29.27% (*n* = 48)	31.71% (*n* = 26)	26.83% (*n* = 22)	0.607
Crawford extent of repair IV	13.92% (*n* = 43)	9.18% (*n* = 9)	16.11% (*n* = 34)	0.101	14.02% (*n* = 23)	10.98% (*n* = 9)	17.07% (*n* = 14)	0.369
Crawford extent of repair V	11.97% (*n* = 37)	13.27% (*n* = 13)	11.37% (*n* = 24)	0.712	14.02% (*n* = 23)	12.2% (*n* = 10)	15.85% (*n* = 13)	0.654
CSF drain	22.19% (*n* = 69)	19.19% (*n* = 19)	23.58% (*n* = 50)	0.385	12.8% (*n* = 21)	17.07% (*n* = 14)	8.54% (*n* = 7)	0.160
HCA	13.83% (*n* = 43)	16.16% (*n* = 16)	12.74% (*n* = 27)	0.415	12.8% (*n* = 21)	14.63% (*n* = 12)	10.98% (*n* = 9)	0.641
Operation time	342 (271–439)	320 (260–409)	346 (278–443)	0.186	319 (258–410)	306 (255–410)	320 (264–411)	0.533
Bypass time	143 (106–211)	142 (105–212)	149 (107–210)	0.509	132 (97–209)	140 (102–214)	130 (93–188)	0.379
Cross-clamp time	102 (72–137)	92 (70–138)	105 (75–137)	0.203	102 (72–140)	92 (73–148)	106 (72–130)	0.347

CSF, cerebrospinal fluid; HCA, hypothermic circulatory arrest.

Statistically significant values are highlighted in bold.

### Postoperative outcome and survival

Postoperative outcome parameters are summarized in Table 3. In this, female patients exhibited a higher rate for postoperative wound infections with an incidence of 12.12% (*n* = 12) compared to 4.25% (*n* = 9) in men (*p* = 0.01). However, no significant differences concerning the observed postoperative outcome parameters were found in the matched cohort. Likewise, the ICU length of stay of 3 days was similar in both groups [3 days (IQR 2–9 days) vs. 3 days (IQR 2–8 days, *p* = 0.979)], and the groups did not differ in the length of postoperative hospital stay.

**Table 3:** Postoperative characteristics.

Characteristics	Unmatched cohort				Matched cohort			
	Overall	Female	Male	*p*-Value	Overall	Female	Male	*p*-Value
In-hospital mortality	21.00% (*n* = 65)	17.3% (*n* = 17)	22.6% (*n* = 48)	0.287	20.7% (*n* = 34)	17.1% (*n* = 14)	24.4% (*n* = 20)	0.248
Respiratory failure	32.15% (*n* = 100)	30.3% (*n* = 30)	33.02% (*n* = 70)	0.633	29.88% (*n* = 49)	28.05% (*n* = 23)	31.71% (*n* = 26)	0.733
Tracheostomy	16.08% (*n* = 50)	13.13% (*n* = 13)	17.45% (*n* = 37)	0.334	16.46% (*n* = 27)	13.41% (*n* = 11)	19.51% (*n* = 16)	0.400
Pneumonia	8.04% (*n* = 25)	9.09% (*n* = 9)	7.55% (*n* = 16)	0.641	10.37% (*n* = 17)	8.54% (*n* = 7)	12.2% (*n* = 10)	0.610
Reintubation	12.54% (*n* = 39)	10.1% (*n* = 10)	13.68% (*n* = 29)	0.375	11.59% (*n* = 19)	10.98% (*n* = 9)	12.2% (*n* = 10)	0.807
Left vocal cord paralysis	3.86% (*n* = 12)	1.01% (*n* = 1)	5.19% (*n* = 11)	0.075	3.05% (*n* = 5)	0% (*n* = 0)	6.1% (*n* = 5)	0.059
Reanimation	3.22% (*n* = 10)	5.05% (*n* = 5)	2.36% (*n* = 5)	0.210	3.66% (*n* = 6)	4.88% (*n* = 4)	2.44% (*n* = 2)	0.682
Sepsis	6.75% (*n* = 21)	6.06% (*n* = 6)	7.08% (*n* = 15)	0.740	5.49% (*n* = 9)	6.1% (*n* = 5)	4.88% (*n* = 4)	0.732
Wound infection	6.75% (*n* = 21)	12.12% (*n* = 12)	4.25% (*n* = 9)	**0.010**	8.54% (*n* = 14)	10.98% (*n* = 9)	6.1% (*n* = 5)	0.403
Rethoracotomy (bleeding)	16.4% (*n* = 51)	13.13% (*n* = 13)	17.92% (*n* = 38)	0.288	13.41% (*n* = 22)	15.85% (*n* = 13)	10.98% (*n* = 9)	0.493
Stroke	3.22% (*n* = 10)	3.03% (*n* = 3)	3.3% (*n* = 7)	0.899	4.27% (*n* = 7)	3.66% (*n* = 3)	4.88% (*n* = 4)	1.000
Temporary paraplegia	2.25% (*n* = 7)	4.04% (*n* = 4)	1.42% (*n* = 3)	0.146	2.44% (*n* = 4)	4.88% (*n* = 4)	0% (*n* = 0)	0.120
Permanent paraplegia	6.43% (*n* = 20)	3.03% (*n* = 3)	8.02% (*n* = 17)	0.095	5.49% (*n* = 9)	2.44% (*n* = 2)	8.54% (*n* = 7)	0.167
Temporary paraparesis	0.96% (*n* = 3)	1.01% (*n* = 1)	0.94% (*n* = 2)	0.955	0.61% (*n* = 1)	1.22% (*n* = 1)	0% (*n* = 0)	1.000
Permanent paraparesis	3.86% (*n* = 12)	2.02% (*n* = 2)	4.72% (*n* = 10)	0.250	3.66% (*n* = 6)	2.44% (*n* = 2)	4.88% (*n* = 4)	0.682
Acute kidney failure	20.58% (*n* = 64)	22.22% (*n* = 22)	19.81% (*n* = 42)	0.624	20.12% (*n* = 33)	21.95% (*n* = 18)	18.29% (*n* = 15)	0.697
Temporary dialysis	6.75% (*n* = 21)	7.07% (*n* = 7)	6.6% (*n* = 14)	0.879	7.32% (*n* = 12)	7.32% (*n* = 6)	7.32% (*n* = 6)	1.000
Permanent dialysis	8.36% (*n* = 26)	10.1% (*n* = 10)	7.55% (*n* = 16)	0.448	7.32% (*n* = 12)	9.76% (*n* = 8)	4.88% (*n* = 4)	0.369
Atrial fibrillation	4.82% (*n* = 15)	6.06% (*n* = 6)	4.25% (*n* = 9)	0.486	7.32% (*n* = 12)	4.88% (*n* = 4)	9.76% (*n* = 8)	0.369
LCOS	5.14% (*n* = 16)	4.04% (*n* = 4)	5.66% (*n* = 12)	0.547	4.27% (*n* = 7)	4.88% (*n* = 4)	3.66% (*n* = 3)	0.699
ECMO	2.57% (*n* = 8)	2.02% (*n* = 2)	2.83% (*n* = 6)	0.674	1.22% (*n* = 2)	2.44% (*n* = 2)	0% (*n* = 0)	0.497
Ventilation time (h)	23.2 (13.9–85.2)	21.1 (13.6–89.7)	23.5 (14.2–83.2)	0.484	19.7 (13.4–82.7)	20.5 (14.0–90.2)	19.7 (12.2–47.5)	0.652
ICU stay time (days)	4 (2–11)	3 (2–8)	4 (2–11)	0.254	3 (2–8)	3 (2–9)	3 (2–8)	0.979
Hospital stay time (days)	14 (11–22)	15 (12–25)	14 (11–20)	0.057	14 (11–20)	15 (12–25)	13 (9–18)	0.100

Statistically significant values are highlighted in bold.

**Figure 2:**
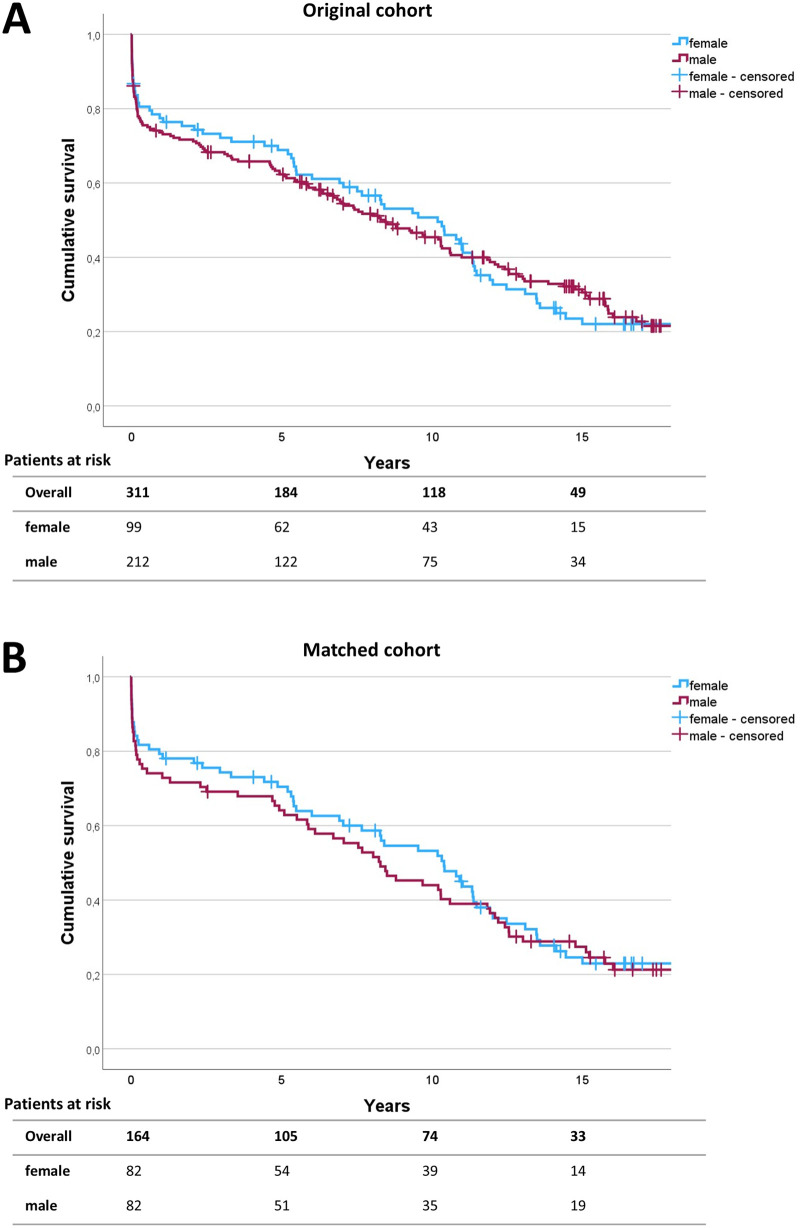
Survival analysis. Survival was analyzed using the Kaplan–Meier survival curves up to 15 years following operation for the unmatched **(A)** and matched **(B)** patient cohort. The numbers of patients at risk are given in 5-year intervals in the follow-up. Log-rank testing revealed *p* = 0.750 for the initial study population and *p* = 0.967 for the matched cohort.

The 30-day mortality rate of female patients was 13.3% (*n* = 13) in the unmatched cohort and 12.2% (*n* = 10) in the matched cohort compared to 16.5% (*n* = 35) and 17.1% (*n* = 14) in the unmatched and matched male cohort, respectively. Long-term follow-up was achieved in 98.4% of the original study population and 98.8% of the matched cohort. Median follow-up time was 6.9 years for the original population and 8.2 years for the matched cohort. No significant differences were found concerning 30-day mortality between men and women in both the unmatched cohort (*p* = 0.463) and the matched cohort (*p* = 0.377). Similarly, no differences were found concerning long-term survival after open thoracoabdominal aortic repair between men and women (Figure 2). The log-rank test revealed *p* = 0.750 for the unmatched cohort and *p* = 0.967 for the matched cohort.

### Risk factor analysis

Risk factor analysis was performed using multivariate binomial logistic regression models for both female and male patients of the initial study population. Hosmer–Lemeshow testing showed sufficient goodness of fit for the models of the male and female cohorts with *p* > 0.05 for both groups. Both models were statistically significant with *p* < 0.001 for each model. With Nagelkerke's *R*^2^ of 0.534, a high amount of explained variance was found for the model of the female subgroup, and with Nagelkerke's *R*^2^ of 0.257, an acceptable amount of explained variance was observed in the model for the male subgroup. In the combined model of the initial study population, the correlation between the key predictor variables and patients sex was <0.8, indicating that confounding interactions between sex and covariates were unlikely.

Concerning the predicting variables of in-hospital mortality of women or men undergoing open thoracoabdominal repair, different parameters were identified as significant predictors for early mortality in female versus male patients (Tables 4A,B). In male patients, cardiac preoperations and the need for urgent repair were identified as risk factors with odds ratios of 2.9 (*p* = 0.022) and 7.3 (*p* = 0.001), respectively. Of the patient inherent characteristics and comorbidity burden, only hyperlipidemia was a significant risk factor with an odds ratio of 1.5 (*p* = 0.006). In contrast, female patients undergoing thoracoabdominal aortic repair exhibited a completely different risk profile compared to males. While the predicting parameters of the male subgroup did not reach statistical significance for prediction of early mortality in the female subgroup, patient inherent comorbidity burden was more predictive for in-hospital mortality among women. Here, hypertension, chronic kidney disease, and the history of coronary artery disease were found to be predictive factors for in-hospital mortality. In contrast to the male subgroup, the age at operation was also found to be a risk factor for early mortality in females.

**Table 4A:** Female risk factors for in-hospital mortality.

Parameter	Odds ratio	95% Confidence interval	*p*-value	Events per variable (deaths/total patients)
Hypertension	3.428	1.378–31.096	0.025	10.2% (*n* = 6 of 59)
CAD	33.134	2.027–514.729	0.014	28.6% (*n* = 6 of 21)
Chronic renal disease	13.126	1.071–160.799	0.044	31.3% (*n* = 5 of 16)
Age at operation	1.637	1.065–2.538	0.028	

LCOS, low cardiac output syndrome; ECMO, extracorporeal membrane oxygenation; ICU, intensive care unit.

**Table 4B:** Male risk factors for in-hospital mortality.

Parameter	Odds ratio	95% confidence interval	*p*-Value	Events per variable (deaths/total patients)
Prior cardiac surgery	2.889	1.167–7.150	0.022	16.1% (*n* = 14 of 87)
Hyperlipidemia	1.469	1.086–5.104	0.006	10.2% (*n* = 6 of 59)
Urgent operation	7.329	2.202–24.369	0.001	31.3% (*n* = 5 of 16)

## Discussion

The main findings of the present study can be summarized as follows: (I) Men and women undergoing open thoracoabdominal repair showed different preoperative comorbidity profiles, with higher rates of cardiovascular and metabolic disorders in men compared to women, while women more often received Crawford Type I repair in the unmatched patient population. (II) Intraoperative parameters, postoperative complication rates, and short- and long-term mortality did not differ significantly between male and female patients. (III) In contrast, men and women had completely different risk factors that were associated with in-hospital mortality.

In the initial study population of the consecutive patients undergoing thoracoabdominal repair at our institution, a higher frequency of Crawford Type I repairs in women (18.4% vs. 9.5%, *p* = 0.027) was observed, which balanced out after propensity score matching. This observation is consistent with prior findings by Spiliotopoulos et al., who reported that women more often undergo less extensive (I and III) thoracoabdominal repairs, whereas men more commonly receive Extent II/IV repairs (8). This suggests an initial sex-based distribution difference that disappears after accounting for baseline characteristics through propensity matching. Moreover, men presented with higher rates of coronary artery disease, hyperlipidemia, and higher BMI compared to women at the time of operation. Similar observations were reported by Latz et al. in patients undergoing Type IV thoracoabdominal aortic repair (15); in that study, men showed significantly higher rates of coronary artery disease compared to women at the time of operation. These findings suggest that male and female patients differ significantly in their individual preoperative risk factor profiles, underlining the importance of propensity score matching for sex-specific analyses in aortic surgery and cardiovascular surgery in general.

In the unmatched cohort, female sex was associated with a higher risk for postoperative wound infections. This contrasts with findings by Chung et al., who investigated postoperative complications in male versus female patients undergoing thoracic aortic surgery (16). Although not reaching statistical significance, they reported a tendency toward higher deep-wound infection rates in men after thoracic aortic surgery compared to women. This discrepancy may be explained by divergent baseline characteristics of the unmatched patient populations in both studies and the limited accuracy of the findings regarding ascending aortic repair via sternotomy versus thoracoabdominal repair via lateral thoracotomy and laparotomy. Moreover, the sex-specific difference observed in our study disappeared after propensity score matching, indicating that the initial disparity was likely driven by baseline differences. Nonetheless, it is noteworthy that postoperative wound infections appeared more frequently in women in the overall study population despite lower incidences of metabolic disorders and lower BMI compared to men.

After propensity score matching, no significant differences were noted between female and male patients in terms of mortality, postoperative complications, operation time, ventilation time, ICU stay, and hospital stay. The currently available literature shows inconsistent findings regarding the influence of sex on postoperative complication rates and survival. The Coselli group reported no significant differences between men and women in short-term mortality and comparable postoperative complication rates in men and women undergoing thoracoabdominal aortic repair (8). Likewise, in a retrospective study of 783 patients undergoing open descending thoracic or thoracoabdominal aortic aneurysm repair by Girardi et al., female sex was not associated with higher early operative mortality compared to the male sex (17). However, women experienced significantly higher rates of postoperative respiratory complications, including increased tracheostomy rates, and had lower 5-year survival in that study. Although more reports on sex-specific outcomes after thoracoabdominal aortic repair are scarce, evidence has to be extrapolated from experiences on thoracic aortic repair. Chung et al. report higher rates of mortality, stroke, and a composite endpoint of stroke, renal failure, deep sternal wound infection, reoperation, and prolonged ventilation in women compared to men (16). Since the work of Chung et al. was a collaborative network analysis and Girardi et al. compared unmatched patient cohorts, both studies were conducted without adjustments for discrepancies in preoperative features in women compared to men. Thus, the described adverse outcome rates in women may have been due to the unequal preoperative burden of comorbidities and age at the time of operation. Contrary to the previous findings, in an experience from complex abdominal aortic aneurysm procedures by de Guerre et al., women had higher perioperative mortality (6.3% vs. 2.4%, *p* = 0.001) and major complications after endovascular aortic repair (EVAR), but no significant sex differences were observed after open aortic repair (18). Similarly, Latz et al. also reported no sex-specific differences in early postoperative mortality and major adverse event rates after open surgical repair of Crawford type I, II, and III aortic aneurysms (19). However, they reported worse long-term survival of women compared to men and identified female sex as an independent risk factor for decreased long-term survival. In contrast, no significant differences in long-term survival were found by Kaplan–Meier analysis in our cohort (Figure 2).

While comparative analyses after propensity score matching revealed no significant differences between men and women regarding postoperative short-term outcome and long-term survival, sex-specific logistic regression models revealed completely different risk factor profiles, contributing to early mortality in men and women. While patient-inherent characteristics in the form of hypertension, coronary artery disease, chronic renal disease, and age at the time of the operation were significant predictors of in-hospital mortality in women, predictors of the male group were hyperlipidemia, urgency, and prior cardiac surgery (Table 4).

The inclusion period between 2000 and 2024 is relatively long, and practice changes over time—particularly concerning perioperative care—may have to be taken into account. However, the rates of female and male patients operated in different time periods were comparable (Supplementary Figure S1). Thus, potential effects due to practice changes would have affected both female and male patients equally. Consequently, it is unlikely that practice changes biased the comparative analyses between female and male patients. Since the rates of chronic dissection and Marfan syndrome did not differ significantly between male and female patients, and only one aortic coarctation was present in a female patient, we conclude that sex-related differences in the aortic pathology are unlikely to bias the sex-specific risk profiles.

The observation that the risk factors for adverse outcomes after thoracoabdominal aortic repair differed distinctly between female and male patients, while the overall postoperative outcomes did not vary between sexes to a significant extent, is consistent with the findings by Spiliotopoulus et al. (8). Their study also reported hyperlipidemia, prior cardiac surgery, and urgent operative indication as significant predictors of in-hospital mortality in male patients. For women, both studies identified age and coronary artery disease as relevant predictors. However, in the present analysis, we demonstrated that hypertension and chronic kidney disease were additional predictors of early mortality in female patients. Spiliotopoulus et al. did not specifically investigate hypertension as a potential predicting variable and found chronic renal insufficiency to be a predicting factor in male but not in female patients.

As the present study is a retrospective analysis, direct conclusions regarding pathophysiological differences underlying the observed differences in risk factors for early mortality cannot be drawn and thus remain speculative. Possible approaches in the era of sex-specific medicine currently emphasize reproductive and hormonal factors as well as autoimmune factors, which could possibly play a role in the context of patients undergoing thoracoabdominal aortic repair. Patient sex and most of the risk factors identified in the current study are already included in the risk evaluation tool EuroSCORE II. However, a sex-specific rating of the diverging factors identified in this study could potentially increase the accuracy of the score system, particularly for female patients.

With chronic renal disease and arterial hypertension as independent risk factors for early mortality in female patients, a thorough nephrological diagnostic workup and, when feasible, treatment may be beneficial, especially for female patients prior to thoracoabdominal aortic repair.

### Limitations

As the underlying study is a retrospective single-center analysis of a highly specialized operation, some inherent limitations apply. The absolute numbers of events per variable in the sex-specific risk factor analysis was relatively low, although the relative event rates per variable were comparatively high. The limited absolute numbers of events per variable result from the limited number of cases included in the study. This is due to the generally very selective and highly specialized nature of the operation, which is performed relatively infrequently, even at a high-volume center such as ours. Moreover, the cause of death in long-term follow-up, especially aortic versus non-aortic death, the and possible contribution of the covariates on overall mortality are unknown in a relevant proportion of the study population, since the vast majority of death occurred in the patients’ homes or peripheral hospitals, where these data are often not obtained or are unavailable.

## Conclusion

While overall outcomes and survival following thoracoabdominal aortic repair were comparable between men and women, this study demonstrates that the underlying risk factors for early mortality differ significantly between sexes. These findings underscore the need for sex-specific preoperative risk assessment in the surgical decision-making process, which is insufficiently addressed in current guidelines. Incorporating sex-specific risk factor stratification strategies may improve patient selection and perioperative management, ultimately leading to better individualized outcomes in complex thoracoabdominal aortic surgery.

## Data Availability

The original contributions presented in the study are included in the article/Supplementary Material; further inquiries can be directed to the corresponding author.

## Supplementary material

Supplementary Figure S1 Time-stratified relative sex distribution. The study population was divided into 5-year time frames with respect to the time of operation. The proportion of male and female patients in the total clientele undergoing surgery during the corresponding periods is shown.
