# Supplementary figures and images for: When similar is not the same: sex-specific outcomes and risk factors in thoracoabdominal aortic repair
Source: Front Cardiovasc Med. 2026 Jan 16;12:1734089. doi: 10.3389/fcvm.2025.1734089 (PMC12856921; doi:10.3389/fcvm.2025.1734089)

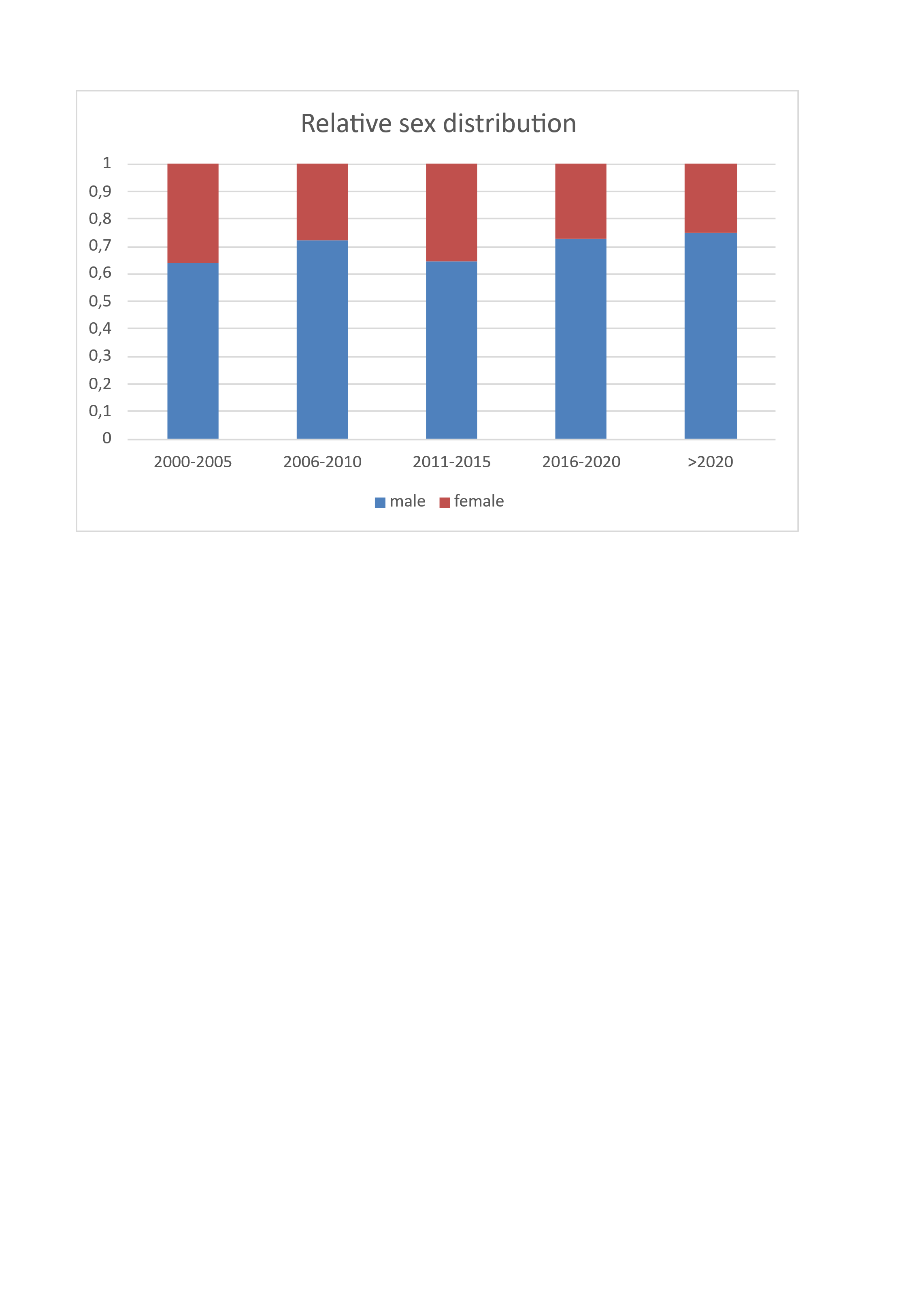

Supplement: Supplementary Figure S1 — Time-stratified relative sex distribution. The study population was divided into 5-year time frames with respect to the time of operation. The proportion of male and female patients in the total clientele undergoing surgery during the corresponding periods is shown. [file Image1.tif]
