# Supplementary material for: When similar is not the same: sex-specific outcomes and risk factors in thoracoabdominal aortic repair
Source: Front Cardiovasc Med. 2026 Jan 16;12:1734089. doi: 10.3389/fcvm.2025.1734089 (PMC12856921; doi:10.3389/fcvm.2025.1734089)
Supplement: Supplementary file 2 [file Table1.docx]

Supplemental Table S1: Parameters for propensity score matching

| Age at operation |
| --- |
| BMI |
| Marfan |
| Hypertension |
| Hyperlipidemia |
| Diabetes |
| Coronary artery disease |
| Cerebrovascular disease |
| Chronic renal disease |
| COPD |
| Tobacco smoking |
| PVD |
| Aortic preoperation |
