# Supplementary material for: When similar is not the same: sex-specific outcomes and risk factors in thoracoabdominal aortic repair
Source: Front Cardiovasc Med. 2026 Jan 16;12:1734089. doi: 10.3389/fcvm.2025.1734089 (PMC12856921; doi:10.3389/fcvm.2025.1734089)
Supplement: Supplementary file 4 [file Table3.docx]

Supplemental table S3: Correlation matrix of the combined binomial regression model

|  | Sex | Coronary artery disease | Hypertension | Chronic renal disease | Hyperlipidemia | Urgent operation | Prior cardiac surgery | Age at operation |
| --- | --- | --- | --- | --- | --- | --- | --- | --- |
| Sex | 1.000 | -0.193 | -0.033 | -0.040 | -0.123 | -0.007 | 0.041 | 0.182 |
| Coronary artery disease | -0.193 | 1.000 | -0.022 | -0.077 | -0.111 | -0.161 | -0.122 | -0.308 |
| Hypertension | -0.033 | -0.022 | 1.000 | -0.143 | -0.203 | -0.019 | -0.158 | -0.152 |
| Chronic renal disease | -0.040 | -0.077 | -0.143 | 1.000 | -0.128 | 0.013 | 0.057 | -0.094 |
| Hyperlipidemia | -0.123 | -0.111 | -0.203 | -0.128 | 1.000 | -0.046 | -0.177 | -0.124 |
| Urgent operation | -0.007 | -0.161 | -0.019 | 0.013 | -0.046 | 1.000 | 0.180 | 0.165 |
| Prior cardiac surgery | 0.041 | -0.122 | -0.158 | 0.057 | -0.177 | 0.180 | 1.000 | 0.315 |
| Age at operation | 0.182 | -0.308 | -0.152 | -0.094 | -0.124 | 0.165 | 0.315 | 1.000 |
